# Prevention of malaria in pregnancy through health education intervention programs on insecticide-treated nets use: a systematic review

**DOI:** 10.1186/s12889-024-17650-7

**Published:** 2024-03-11

**Authors:** Opara Monica Onyinyechi, Suriani Ismail, Ahmad Iqmer Nashriq Mohd Nazan

**Affiliations:** Department of Community Health, Faculty of Medicine and Health Sciences, Universiti Putra Malaysia, UPM Serdang, Serdang, Selangor 43400 Malaysia

**Keywords:** “Malaria”, “Malaria prevention”, “Health education”, “Insecticide-treated nets”, “Utilization”, “Pregnant women”

## Abstract

Malaria is a widespread and prevalent disease that affects human population globally, particularly in tropical countries. Malaria is a major health issue in sub-Saharan Africa and it contributes to morbidity and mortality among individuals in Africa. Pregnant women have been also reported as high risk of people been infected with malaria. This review attempted to evaluate the various methods used for health education programs and the effectiveness of the programs in improving ITNs among pregnant women.

**Methods** The search involved various databases; EBCOHOST, MEDLINE, CINAHL, Cochrane library, ScienceDirect, PubMed, SAGE, Sringer link, Web of Science and Wiley Online Library. It was limited to full text research articles that report intervention studies, written in English Language, published between 2003 to 2022. The key words were “malaria”, “malaria prevention”, “health education”, “insecticide-treated nets”, “utilization”, “pregnant women”.

**Results** A total of eleven articles met the inclusion criteria and included in the review. Six studies reported randomized controlled trials (RCTs) while five reported non-randomized controlled trials (NRCT).

**Conclusions** There are evidences from the results which showed that health education programs were improved among pregnant women due to the use of ITNs and LLINS utilization. Furthermore, additional interventions directed at significant others need to be implemented, considering their important role in determining pregnant women’s use of ITNs.

## Introduction

Malaria is a widespread and persistent disease that affects humans’ inhabitants globally, particularly in tropical countries. It is among the major health problems in sub-Saharan Africa and it contributes to morbidity and mortality among individuals in Africa [1]. According to what was well-described previously, the incidence of malaria decreased steadily from about 2000 to 2015, with delaying of progress since that time. There were 241 million cases of malaria in 2020 and an increase of 6% from 227 million in 2019 which WHO estimated for at latest (which, notably, include changes in estimates for past years) [2]. The distribution of mortality in young children markedly raised an estimates of malaria deaths that included a change for the past years. In 2020, deaths attributed to malaria increased to 627,000 as compared with 558,000 in 2019, 562,000 in 2015, and 896,000 in 2000. With the new baseline, it is estimated that 47,000 of the 69,000 increased deaths, compared with those in the previous year. In 2020, the results of service were disrupted due to the Covid-19 pandemic [2]. The problem has recently been worsened by the challenges of the pandemic and it made the progress against malaria to be stalled. The malaria problem has been greatest in Africa through recent times, but the imbalance between Africa and the rest of the world has been growing. Recently, many countries outside African have seen a remarkable malaria profits with so many moving towards elimination. In 2021, WHO certified China free from malaria. Meanwhile in 2020, sub-Saharan Africa accounted for 95% of the malaria burden [3]. Six countries in Africa accounted for 55% of cases and the countries included were Nigeria, Democratic Republic of the Congo, Uganda, Mozambique, Angola, and Burkina Faso. Malaria can be appreciated as primarily an African problem as the burden is overwhelmingly from Plasmodium falciparum; Plasmodium vivax, which is little seen in most of Africa, now makes up only 2% of total global cases even though the problem keeps persisting in a large part of the tropics [2]. Most of the global population lives in areas were malaria is endemic, pregnant women and young children below five years are vulnerable group for malaria infection [4]. A total of 10,000 pregnant women and 200,000 children die due to the complications of malaria annually [5]. An estimated of 207 million cases had led to around 627,000 mortality in 2012 [6]. In malaria endemic areas more than half of pregnant women are predictable to be asymptomatic carriers of parasitaemia [7]. Pregnant women have been also reported as high risk of people been infected with malaria. Malaria during pregnancy is associated with numerous health issues, this include decreases level of haemoglobin, miscarriage and premature delivery [8–10].

The WHO recommended three approach to malaria control during pregnancy which consist of the use of insecticide-treated nets (ITNs), intermittent preventive treatment (IPT), and case management treatment [11] *and the pregnant women are encouraged to take preventive treatment monthly.* Sleeping under an ITN each night and taking two doses of IPTp with sulfadoxine-pyrimethamine (SP) in pregnancy have been confirmed to reduce malaria infection risk and its complications in pregnancy [12–14].

Although trials have established that ITNs are effective malaria control approach. Within the period of 2019–2020, about 590 million ITNs were delivered to communities in sub-Saharan Africa, where most ITNs are distributed. However, the estimated percentage of the population with access to an ITN within their household and the percentage of the population sleeping under an ITN was 54% and 47%, respectively in 2021 which owned to several reasons.

 [15]. In some parts of Africa, it has been previously reported that lack of access to ITNs and poor knowledge and perception on ITNs and malaria is a great important barrier to the use [16]. Though, access does not always result in usage due to sociocultural and logistical reasons [17, 18] reported that over 90% of its respondents found ITNs to be uncomfortable to use, especially during pregnancy. The persistence of malaria challenges is predominantly felt in Africa, largely attributed to the setbacks caused by the disruptions of the COVID-19 pandemic. These disruptions have hampered the strides made in the efforts to eliminate the disease. Even with much studies on ITNs use, pregnant women still get infected with malaria during pregnancy. Use of insecticide treated nets in pregnancy has remained poor in spite of increased health education and awareness campaign by government agencies (Ezeama et al., 2014) (study gap). Thus, this paper reviews literature in order to understand the impact of health education intervention on the effects of ITNs in malaria prevention among pregnant women. Challenges of malaria burden still remain mostly in Africa due to the disruption related to COVID-19 pandemic which has set back all the progress that has been put in eradicating the disease. However, research on malaria still remains very much active by leading to the most important new tools to control the hardest hit areas which will make us to move towards eliminating the disease in many countries. There is an increased worldwide attention on the control of infectious diseases. We hope in a coming year, this attention will outgrowth will improve the efforts to eliminate and control malaria.

The purpose of this review is to evaluate the various methods used for health education programs and its effectiveness in improving the use of ITNs among pregnant women.

## Materials and methods

**Fig. 1:**
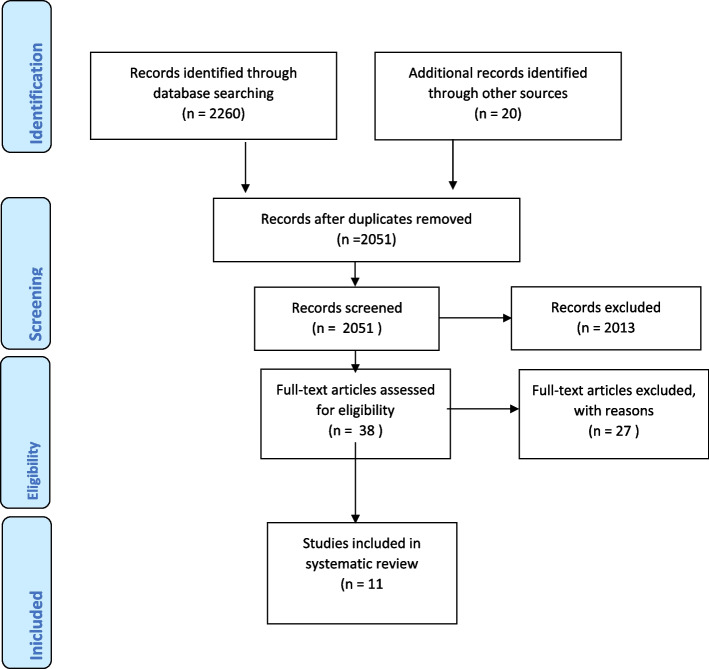
PRISMA flow Diagram

The Preferred Reporting Items for Systematic Reviews and Meta-analyses (PRISMA) guidelines was used to report this review Fig. 1 (Boutron, Moher, Altman, Schulz & Ravaud). Inclusion criteria in this review were restricted to Health education intervention studies on insecticide-treated nets utilization among pregnant women, for example, randomized control trials (RCTs) and non-randomized controlled study (NRS) assessing the effects of health education interventions for pregnant women. Age limits of participants from 18 years and above. The intervention includes educational programs involving teaching, discussion, practical and demonstration. Exclusion criteria were articles that are Non-experimental studies, review papers, pharmacological trials, studies that focus on other chronic diseases were excluded. Articles that are not published in English language and articles published before 2003. Health education interventions studies that did not focus on malaria prevention in pregnancy, and studies that malaria prevention education are not sole intervention were excluded. The search involved various databases; EBCOHOST, MEDLINE, CINAHL, Cochrane library, ScienceDirect, PubMed, SAGE, Sringer link, Web of Science and Wiley Online Library. It was limited to full text research articles that reported only intervention studies that were written in English Language and it was published between the year 2003 to 2022. The key words used in the search were “malaria”, “malaria prevention”, “health education”, “insecticide-treated nets”, “utilization”, “pregnant women”.

Reference lists were checked and search for important studies, in order to detect additional related publications. Independently two authors reviewed the full-text articles to check if inclusion criteria were in accordance and compared results at each stage. All article retrieved during the search was assessed independently by two authors of the team. Each article titles and abstracts were screened subsequently and the full text screening was reviewed by two authors.

### Search outcome

Studies on educational programs to improve insecticide-treated nets utilization for prevention of malaria among pregnant women were searched. A total of 2260 studies were identified through electronic searching using key words. Each article title and abstract were initially reviewed and assessed to know if they correspond with inclusion criteria to review the full text, published review paper, abstract, conference paper, dissertation and thesis were excluded from this review. Only 38 articles were included to review in full text. Only articles on Randomized Control Trails (RCTs) and Non-Randomized Studies (NRS) designs were included. Among the 38 articles, a total of 27 articles were excluded because of these reasons: articles are not intervention study and articles that are protocol development. The studies reviewed were based ITNs use in malaria prevention, pregnant women, health education programs and follow-up.

"The Consolidated Standards of Reporting Trials (CONSORT) statement for assessing non-pharmacologic treatments checklist was used as a reporting guideline to evaluate the articles" [19, 20] Table 1. The CONSORT components covered are title, abstract, introduction, methodology, results and discussion.

**Table 1:** Health education interventions to improve usage of ITNS, LLITNs among pregnant women

Author	No of participants	Age of participants	Study type	Intervention type	Study country	Outcome	Findings
Balami et al. (2021) [21]	372	15 to 45 years	Randomized Controlled Trial	Health education given at a group level, and in a single session	Nigeria	The effects of group, time, and group-time interaction on reported ITN use and IPTp uptake, as well as haematocrit level, were all significant	The intervention was effective in improving ITN use, IPTp uptake, and haematocrit levels
**Adeniji et.al. (2023) ** [22]	**90**	**Not mentioned**	**Quasi-experimental design**	**Health education given at a group level, and in a single session**	**Nigeria**	**Examine the attitude towards malaria prevention among pregnant women at immediate post intervention for experimental and control groups**	**Findings from this study increased the importance of health education as a potential tool in health campaigns to improve attitude towards malaria prevention among patients**
Deribew et al. (2010) [23]	442	15 to 45 years	Randomized Controlled Trial	Tailored training on the proper use of ITNs and establishing community network system	Ethiopia	The availability and utilization of ITNs was low in the study area	The utilization of ITN was hampered by lack of awareness in the rural community who lives in traditional Tukul houses
Elmosaad, Y. M., et.al. (2016) [19]	761	Not mentioned	Randomized Community Trial	Health education given at a group level, and in a single session	Sudan	The communication for behavioural impact strategy in enhancing the use of LLITNs	The intervention was effective in improving LLITN use. It is recommended that there is a need to concentrate on communication for behavioural impact strategies to enhance utilization of LLITNs through all available channels
**Melo et al. (2018) ** [24]	**215**	**Not mentioned**	**Randomized Controlled Trial**	**Effectiveness of an action-oriented educational intervention in ensuring long term improvement of knowledge, attitudes and practices of community health workers in maternal and infant health: a randomized controlled study**	**Brazil**	**There is an increase and retention in the average overall KAP score at one-year follow up as compared to baseline and to control group and the secondary outcomes increased in the average score after the health education training**	**The intervention group showed significant improvements with respect to baseline in overall KAP score as well as in K and P scores. In the control group, overall KAP as well as separate K, A and P scores remained unchanged between baseline and follow up assessments**
**Amoran et al. (2012) ** [25]	**400**	**Not mentioned**	**quasi-experimental design**	**Health education was given at a group level**	**Nigeria**	**The impact of health education increases the use of ITNs**	**The use of ITN in the study population was significant increase by health education. It also demonstrated the effect of health education on improved correct knowledge and attitude about ITN. Uptake of ITN can be significantly improved in rural areas if the nets are made available and backed up with appropriate health education intervention**
Kumar et al. (2020) [26]	200	18 and 45 years	Quasi-experimental, design	Health education given at a group level	Pakistan	The effectiveness of health education on the adoption of ITNs among pregnant women	Health education could be an effective intervention for improving knowledge and usage of ITNs among pregnant women for the prevention of malaria
Ahmadi et al. (2012) [27]	Not mentioned	15 years and above	Intervention community-based cross-sectional survey	The intervention groups were educated together as units	Iran	The use of ITNs among mosquito net owners, and also generate information on knowledge and practices regarding malaria and it’s preventive measures in general, and ITNs use	A significant increase was seen in the proportion of households who used ITNs the previous night (92.5%) compared with untreated nets (87.1%)
Okeibunor et al. (2011) [28]	1,280	15–49 years	Quasi-experimental, design	Deliver ITNs to pregnant women in the treatment arm communities	Nigeria	increase effective access to malaria prevention among pregnant women; specific targets were increasing ITN use, and increasing access to IPTp in the form of two doses of SP during pregnancy	women in the control area, an additional 7.4% of women slept under an ITN during pregnancy in the treatment areas
Ahmed et al. (2011) [29]	Not mentioned	Not mentioned	Intervention community-based cross-sectional survey	Health education was given in households. During the households visit education on malaria and its transmission, education on the norms of use of insecticidal treated nets (e.g., when to hang the net, where and how to wash and dry the net, and how many washes in a year etc.). Education on diagnose (by rapid diagnostic test, RDT) and to treat malaria promptly	Bangladesh	ITN, and at least one ITN ownership	There was significant improvement in the proportion of pregnant women who slept under an insecticidal bed net in 2011 compared to 2008, in both areas. ITN, and at least one ITN ownership increased (22–59 to 62–67% and 22–64% to 74–76% respectively) over time. substantial improvement in possession and usage of insecticidal bed nets
Rhee et al. (2011)	86	14 years and above	Controlled intervention study	Health education was given regarding signs and symptoms of malaria, susceptibility, transmission and prevention of malaria and information about the benefits of ITN use, including, how, when and where to impregnate nets	Mali	The main outcome measure was ITN use	ITN use was significantly greater in participants who had received the educational intervention (48%) vs. individuals who did not (33%)

Abstracts was reviewed and screened by one author for inclusion criteria. Second set of reviewers assessed the retrieved articles for uncertainty. Articles that met the inclusion criteria were included to review full-text to confirm if inclusion criteria were met. Cochrane Risk of Bias Tool was applied by the authors to assess risk of bias, Table 2 shows summary of risk of bias (Cochrane Statistical Methods Group and the Cochrane Bias Methods Group). Based on the tool, assessing risk of bias in studies were as follows: "random sequence generation allocation concealment, blinding of participants and personnel, blinding of outcome assessment, incomplete outcome data, short-term outcomes (2–6 weeks), incomplete outcome data, long-term outcomes (> 6 weeks) and selective reporting [30].

**Table 2:** Risk of bias summary (Cochrane Statistical Methods Group and the Cochrane Bias Methods Group)

Author	Random sequence generation (selection bias)	Allocation concealment (selection bias)	Blinding of Participants and personnel (performance bias	Blinding of outcome assessment (detection bias) (patient-reported outcomes	Blinding of outcome assessment (detection bias) (Mortality)	Incomplete outcome data addressed (attrition bias) (Short-term outcomes (2–6 weeks)	Incomplete outcome data addressed (attrition bias) (Longer-term outcomes (> 6 weeks)	Selective reporting (reporting bias
Balami et al. (2021) [21]	+	+	+	+	+	+	+	+
Adeniji et.al. (2023) [22]	+	+	+	+	+	+	+	+
Deribew et al. (2010) [23]	+	+	+	?	?	?	?	+
Elmosaad, Y. M., et.al. (2016) [19]	+	+	+	-	?	-	-	?
Melo et al. (2018) [24]	+	+	_	_	_	_	_	+
Amoran et al. (2012) [25]	+	_	_	_	?	_	+	+
Kumar et al. (2020) [26]	+	+	_	_	_	_	_	+
Ahmadi et al. (2012) [27]	+	?	_	_	_	_	_	_
Okeibunor et al. (2011) [28]	+	?	_	_	_	_	_	+
Ahmed et al. (2011) [29]	+	?	_	_	_	_	_	+
Rhee et al. (2011)	+	?	_	_	_	_	_	+

High Risk (^_^)

Low Risk ( +)

Unclear risk (?)

## Results

In this systematic review, a total of 463 records were retrieved from the databases based on our search strategy, 11 studies were reviewed (Table 1). The revived studies reported the sample size and it is from 25 to 495 patients. All studies stated respondents age and the age ranged from 18 years and above. Based on our risk assessment, (Table 2) summarized the risk of bias for the reviewed studies. Among the reviewed studies, the risk of bias was not clear however, two studies reported methodologically low risk of bias [21, 31]. For both studies, the education interventions improved insecticide-treated nets utilization among pregnant women. The possible risk of bias is an evidence selection bias such as: random sequence generation, allocation, performance etc. (kindly check Table 2 for easy understanding).

### Types of intervention studies

Six studies reported randomized controlled trial (RCTs) [21–24, 31, 32] and Five studies reported non-randomized controlled trial (NRCT) [25–29].

Most studies focus on education programmes on insecticide-treated nets utilization and its effectiveness over other prevention technique. Education programmes in all the studies was taught by trained personnel that has knowledge of research and they are either medical or health science graduates. Among the reviewed articles, studies were carried out either in health facility or at participant’s house. Some studies reported that there was no control group in their studies. Regarding the study location, eight studies were carried out in health facilities [21, 23–27, 31, 32].

One study was conducted in household [28]. One study was conducted in Research Centre [22]. The educational approach included one-to-one or group approaches, groups discussion, teaching delivery method, demonstration on use of ITN posters and manuals.

A study reported that the intervention group received a four-hour health education intervention on ITN use, while the control group received a similar designed health education on breastfeeding [31]. Another study mentioned that pregnant women in the intervention group received health education sessions on malaria for 12 weeks, while those in the control group received routine information from health workers. Pre- and post-intervention assessment was on knowledge regarding malaria and use of insecticide bed nets [25]. In another study, the intervention group received health education on malaria, while the control group received health education on breastfeeding by the same facilitator [21]. Another study reported that health education and training was given to intervention group and control group on how to use and hang the bed net [22]. Meanwhile five studies did not mention what was given to the control group [23, 26–28, 32]. However, one study did not mention what was given to the intervention group and the control group [24]. Follow-up time differs in various studies, some was assessed at baseline. There was an observed time interval difference from baseline to end of the research in the reviewed studies. The longest evaluation time of two years follow‑up. One study follow-up at patients’ home [24].

### Outcome measures

In the reviewed studies, outcomes measured were total knowledge, motivation and behavioural skills scores among pregnant women. Perceptions and practices regarding malaria and to improve ITNs use, ensure usage of ITNs among pregnant women. The reduction in severe anaemia at delivery, the reduction in low birth weight. The level of knowledge about malaria, malaria prevention among pregnant women; specific targets were increasing ITN use, and increasing access to intermittent preventive treatment (IPTp) in the form of two doses of sulphadoxine-pyrimethamine (SP) during pregnancy.

In the studies reviewed, a study pointed out that the intervention had significantly improved ITN use for the intervention group and also IPTp uptake at second follow up increased in the intervention group [31]. Moreover, a study improved in scores of knowledge and increase in use of LLINs scores in the intervention group compared to control after the intervention which was significant, and this shows that the intervention program had a positive effect [25]. Another study showed improvement in knowledge of ITN use, motivation, and behavioural skills scores respectively, for the intervention group over the control group [21]. Another study reported that the significant increase in the proportion of households who used LLINs the previous night compared with untreated nets participant. The study also reported that the educational program increased respondent’s knowledge regarding malaria transmission in intervention and control group. Also, respondent’s knowledge regarding mosquitoes breeding places improved in both intervention and control group. Knowledge regarding critical time to hang the net also increased over time in both intervention and control group but it remained quite low [26]. A study reported that there were improvements for ITN use for both intervention group and control group, however there was increases use of ITN in intervention group compared to control group. The education intervention program also improved adherence of IPTp and also increased the fraction of pregnant women that took minimum of two SP doses during pregnancy [27]. A study shows improvement in utilization of ITN in the control group when compared to the intervention group [22]. Another study shows improvement in ITN’s use for both the intervention and control groups for more than 90% of the participants improved in ITN’s use [23]. A study revealed that a significant increase also was seen in the proportion of households who used ITNs the previous night compared with untreated nets. Educational status was an important predictor of ITNs use. Regular use of ITN among the respondents were considered higher than the targeted coverage (80%) which was recommended by World Health Organization (29). In another study, the intervention group got improved due to the use of ITNs and household ownership of ITNs increased significantly over the study period with a significantly higher increase in the intervention group as compared to control group between the baseline and follow-up (24). A study revealed that in intense malaria transmission areas, ITN decrease adverse effect of malaria during pregnancies [24].

## Discussion

This review reported 11 studies on education of malaria preventive measures among the pregnant women from different kinds of intervention including RCTs and NRSs. Discussion method was based on the reviewed articles, the NRSs has high risk of bias, although the studies are relevant and have information on health education programme. Malaria prevent education among pregnant women which comprises of ITNs, LLINs IPTp, SP during pregnancy, written or spoken instructions on malaria prevention, group discussions on malaria prevention and also counselling that focused on promoting ITNs, aiming at preventing malaria in pregnancy.

WHO currently recommends that pregnant women in Africa malaria endemic region should use both IPTp-SP and ITNs for malaria prevention, the trials assessed the effect of ITNs and IPTp-SP simultaneously, the results showed that ITNs provided benefits in primigravidae if used alone [33, 34]. The result of the reviewed studies shows that all education programs applied by the previous researchers’ shows improvement on ITNs, LLINs, IPTp, SP, knowledge of malaria transmission in intervention and control group. Also, respondent’s knowledge regarding mosquitoes breeding places among the pregnant women. Among the review articles, trained personnel in research field delivered educational programs and this will prevent observer bias.

Some studies were carried out in health facility and some at participants house based on the reviewed articles. With regards to the methods of educational programs, different intervention studies used different methods and approaches. The education programs were carried out in group session or one on one method, this include discussions, counselling, demonstrations. Discussions method is more effective because it helps to express, clarify participants knowledge, experiences and feelings. Discussion method helps the participants to apply and interchange ideas within the group. However, the challenges in discussion method is that it is time consuming, this is as a result of allocated time for the participants to ask questions after the discussion. Discussion method is not expensive unlike other methods example is demonstration method which requires materials to teach the respondents.

Among the eleven articles in this review only two studies were low risk of bias [21, 31]. Both studies reported that the education interventions improved ITN use for the intervention group and also IPTp uptake at second follow up increased in the intervention group, also for the intervention group, there was improvement in knowledge of ITN use, motivation, and behavioural skills scores respectively.

In evaluating program effectiveness, Randomized Controlled Trials (RCTs) emerge as a superior design, offering a direct exploration of cause-and-effect relationships with minimal bias [35]. The straightforward nature of RCTs facilitates the investigation of program impact compared to observational studies, with the added advantage of easy blinding/masking (Jones, 2018). This characteristic enhances the reliability of findings, contributing to the validity of the study.

Moreover, the analysis of RCT results is streamlined through the utilization of well-established statistical packages, enhancing the robustness of the conclusions drawn [36]. Clearly defined populations of participating individuals in RCTs contribute to the transparency of the study, allowing for precise identification of contributing factors [37].

In light of these strengths, the authors of this paper advocate for the consideration of RCTs in future studies, particularly for long-term follow-ups [38]. Emphasizing patient-centered interventions, powered samples, strategic randomization approaches, and meticulous concealment and reporting of sample information are crucial for the success of future RCTs [39].

Furthermore, the authors recommend focusing on research that evaluates educational programs effective in improving Insecticide-Treated Nets (ITNs) and Long-Lasting Insecticidal Nets (LLINs) use among pregnant women. This emphasis on high-quality RCT design is paramount, as it ensures the generation of robust recommendations for healthcare practitioners and clinicians regarding optimal educational interventions to prevent malaria [40]. The impact of such interventions can potentially be transformative, contributing significantly to public health efforts in malaria prevention.

### Strength and limitations of the study

This review reports the finding from studies that focus on effectiveness of health education intervention programs to improve ITNs, LLINs use among pregnant women. The selected articles were original research only, written in English language from year 2003 to 2022. Selection bias may occur during data extraction, due to only full text articles were searched. This review adherence with proper systematic review methodology, however because of limited time, resources and methodological issues in some of the reviewed studies, the authors could not proceed to meta-analysis.

## Conclusion

From the 11 reviewed studies, there was a higher knowledge and practice of insecticide treated use among the intervention group during the intervention and the final follow-up visits. We can conclude in this review that the intervention program was effective in improving the study outcomes. The use of ITNs and LLINs is important and will also help for decision-making in the national malaria control program campaigns. Due to the ease of its implementation at scale, it can also improve maternal morbidity and mortality which could not only be beneficial in preventing malaria and reducing its burden of disease. Furthermore, additional interventions directed at significant others need to be implemented, considering the important role they play in determining pregnant women’s use of insecticide treated nets.

## Data Availability

This is purely a review paper, there was no data/analysis. The discussions and conclusions are purely based on the papers reviewed. Anyone that wishes to get the study data should contact the “Corresponding author”.
